# MXRA5 is a TGF‐β1‐regulated human protein with anti‐inflammatory and anti‐fibrotic properties

**DOI:** 10.1111/jcmm.12953

**Published:** 2016-09-06

**Authors:** Jonay Poveda, Ana B. Sanz, Beatriz Fernandez‐Fernandez, Susana Carrasco, Marta Ruiz‐Ortega, Pablo Cannata‐Ortiz, Alberto Ortiz, Maria D. Sanchez‐Niño

**Affiliations:** ^1^ IIS‐Fundacion Jimenez Diaz Universidad Autonoma de MadridREDINRENMadridSpain; ^2^ School of MedicineUniversidad Autonoma de MadridMadridSpain; ^3^ Fundacion Renal Iñigo Alvarez de Toledo‐IRSINMadridSpain

**Keywords:** chronic kidney disease, fibrosis, kidney, inflammation, polycystic kidney disease, perlecan, adlican

## Abstract

Current therapy for chronic kidney disease (CKD) is unsatisfactory because of an insufficient understanding of its pathogenesis. Matrix remodelling‐associated protein 5 (MXRA5, adlican) is a human protein of unknown function with high kidney tissue expression, not present in rodents. Given the increased expression of MXRA5 in injured tissues, including the kidneys, we have suggested that MXRA5 may modulate kidney injury. MXRA5 immunoreactivity was observed in tubular cells in human renal biopsies and in urine from CKD patients. We then explored factors regulating MXRA5 expression and MXRA5 function in cultured human proximal tubular epithelial cells and explored MXRA5 expression in kidney cancer cells and kidney tissue. The fibrogenic cytokine transforming growth factor‐β1 (TGFβ1) up‐regulated MXRA5 mRNA and protein expression. TGFβ1‐induced MXRA5 up‐regulation was prevented by either interference with TGFβ1 activation of the TGFβ receptor 1 (TGFBR1, ALK5) or by the vitamin D receptor agonist paricalcitol. By contrast, the pro‐inflammatory cytokine TWEAK did not modulate MXRA5 expression. MXRA5 siRNA‐induced down‐regulation of constitutive MXRA5 expression resulted in higher TWEAK‐induced expression of chemokines. In addition, MXRA5 down‐regulation resulted in a magnified expression of genes encoding extracellular matrix proteins in response to TGFβ1. Furthermore, in clear cell renal cancer, von Hippel–Lindau (VHL) regulated MXRA5 expression. In conclusion, MXRA5 is a TGFβ1‐ and VHL‐regulated protein and, for the first time, we identify MXRA5 functions as an anti‐inflammatory and anti‐fibrotic molecule. This information may yield clues to design novel therapeutic strategies in diseases characterized by inflammation and fibrosis.

## Introduction

Chronic kidney disease (CKD) is defined as abnormalities of kidney structure or function, present for more than 3 months, with implications for health 1. Indeed, CKD is among the fastest growing causes of death worldwide and is a major cause of disability [2, 3]. Mortality results both from accelerated cardiovascular disease and from progression to end‐stage renal disease (ESRD). ESRD patients require lifelong dialysis or kidney transplantation and have a mortality which is 10‐ to 100‐fold higher than the general population 4. Renal inflammation and fibrosis are key pathological processes involved in the progression of CKD to ESRD. Current therapy for CKD is unsatisfactory and has failed to prevent the increase in CKD‐associated mortality. Anti‐proteinuric agents targeting the renin–angiotensin system have been the backbone of kidney disease therapy for almost 30 years 5. However, as exemplified by diabetic nephropathy, the most common cause of ESRD worldwide, promising new drugs usually fail key phase 2 or phase 3 trials 6. Thus, an improved understanding of the pathogenesis of CKD and, specifically, of the molecules governing the transition to chronicity is needed in order to design novel therapeutic approaches to CKD 7.

Inflammation is a key promoter of CKD progression, eventually leading to renal fibrosis. Renal fibrosis arises from imbalances in extracellular matrix (ECM) homoeostasis, leading to ECM accumulation. Renal fibrosis is the strongest histological predictor of CKD progression, regardless the cause of the primary disease 8. Among the multiple factors contributing to kidney inflammation and fibrosis, agents targeting the inflammatory cytokine TWEAK and the fibrogenic protein TGFβ1 have reached the clinical trial stage, albeit not yet successfully [9, 10]. TWEAK activation of Fn14 promotes podocyte injury 11, tubulointerstitial inflammation 12 and interstitial fibrosis 13, while TGFβ1 promotes fibrosis and dampens inflammation, promoting the scarring process as resolution of inflammation.

Matrix remodelling‐associated protein 5 (MXRA5), also known as adhesion protein with leucine‐rich repeats and immunoglobulin domains related to perlecan (Adlican), is a protein of unknown function belonging to the MXRA gene family. The MXRA5 gene encodes a protein of a predicted molecular weight of 312 kDa. The MXRA family comprises three genes (MXRA5, MXRA7 and MXRA8) and is thought to participate in cell adhesion and matrix remodelling 14. MXRA5 is found in primates, marsupials, some mammals, birds and fishes, but not in mouse or rat. MXRA5 mRNA is up‐regulated in human chronic ischaemic myocardium, together with other genes promoting ECM remodelling 15 and in fibroblast primary cultures from centenarians 16. Although MXRA5 functions are not well known, these data point to a potential role in tissue injury and fibrosis. The kidney is among the highest MXRA5 expressing organs (http://www.ncbi.nlm.nih.gov/IEB/Research/Acembly/av.cgi?db=human%26q=MXRA5; accessed May 15, 2016). Thus, we hypothesized that MXRA5 may play a role in kidney inflammation or fibrosis and we now report on the factors regulating MXRA5 expression in kidney tubular cells and, for the first time, on MXRA5 function.

## Materials and methods

### Ethics

The IIS–Fundación Jiménez Díaz Biobank is part of the ISCIII Network of Biobanks. The biobank and biobank consent forms have been approved by the Ethics Committee of the IIS‐Fundación Jiménez Díaz and patients who donated samples signed an informed consent form. The current project was also approved by the Ethics Committee of the IIS‐Fundación Jiménez Díaz and conforms to the Declaration of Helsinki and current Spanish legislation.

### Cells and reagents

HK‐2 human proximal tubular epithelial cells (ATCC, Rockville, MD, USA) were grown on RPMI 1640 (Life Technologies, Grand Island, NY, USA) with 10% heat‐inactivated FBS, 2 mM glutamine, 100 U/ml penicillin, 100 μg/ml streptomycin, 5 μg/ml insulin, 5 μg/ml transferrin, 5 ng/ml sodium selenite and 5 ng/ml hydrocortisone in 5% carbon dioxide at 37°C 17. VHL‐defective clear cell renal carcinoma cells (VHL−/− ccRCC) and VHL+/+ ccRCC cells have been previously described 18. For experiments, cells were rested in serum‐free media 24 hr prior to the addition of stimuli and throughout the experiment. Five hundred thousand cells were seeded in 60 mm diameter wells for RNA extraction, Western blot or flow cytometry experiments. Cells were treated with 100 ng/ml TWEAK (Millipore, Watford, UK), 1 ng/ml TGFβ1 (Peprotech, London, UK), 100 nmol/l paricalcitol (Abbot, Chicago, Illinois), 100 μmol/l TGFβ1 receptor 1 (ALK5) inhibitor (SB431542; Sigma‐Aldrich, Sigma, St. Louis, MO) or 1 ng/ml neutralizing anti‐TGFβ1 antibody (ab100NA; R&D Systems, Minneapolis, MN), based on prior dose–responses studies from our lab.

### Transfection of small interfering RNA

Cells were grown in six‐well plates (Costar, Cambridge, MA, USA) and transfected with a mixture of 30 nmol/ml MXRA5 siRNA (Santa Cruz Biotechnology, Santa Cruz, CA, USA), Opti‐MEM I Reduced Serum Medium and Lipofectamine RNAiMAX Transfection Reagent (Invitrogen, Life technologies, Paisley, UK). After 18 hr, cells were washed and cultured for 48 h in complete medium and serum‐depleted for 24 hr before stimulation. A scrambled siRNA (Ambion, Applied Biosystems, Foster City, CA, USA) was used as control.

### RNA extraction and real‐time polymerase chain reaction

Total RNA was extracted from cells by the TRI‐Reagent method (Sigma‐Aldrich) and 1 μg RNA was reverse transcribed with High Capacity cDNA Archive Kit (Applied Biosystems). Pre‐developed primer and probe assays for MXRA5, GAPDH, MCP‐1, RANTES, CXCL16, Fibronectin, Type IV Collagen and 18S were from Applied (Applied Biosystems). Quantitative PCR was performed by 7500 Real‐Time PCR System with the Prism 7000 System SDS Software (Applied Biosystems) and RNA expression of different genes was corrected for GAPDH or 18S 12.

### Western blot

Cell samples were homogenized in lysis buffer (50 mM Tris–HCl, 150 mM NaCl, 2 mM EDTA, 2 mM EGTA, 0.2% Triton X‐100, 0.3% NP‐40, 0.1 mM PMSF and 1 μg/ml pepstatin A) and then separated by 15% SDS‐PAGE under reducing conditions. After electrophoresis, samples were transferred to PVDF membranes (Millipore), blocked with 5% BSA in PBS/0.5% v/v Tween 20 for 1 hr, washed with PBS/Tween, and incubated with goat polyclonal anti‐MXRA5 (1:500 in 1% BSA PBS/Tween; Santa Cruz Biotechnology). Blots were washed with PBS/Tween and incubated with appropriate horseradish peroxidase‐conjugated secondary antibody (1:2000, Santa Cruz Biotechnology). After washing with PBS/Tween, blots were developed with the chemiluminescence method (ECL) (Amersham). Blots were then probed with mouse monoclonal anti‐α‐tubulin antibody (1:10,000; Sigma‐Aldrich) and levels of expression were corrected for minor differences in loading 19.

### Immunohistochemistry

Immunohistochemistry was carried out in paraffin‐embedded human kidney, fixed in 4% buffered formalin. The primary antibody was goat polyclonal anti‐MXRA5 (1:100; Santa Cruz biotechnology). Briefly, 5‐μm‐thick renal sections were adhered to polylysine‐coated glass slides and fixed overnight at 56°C. After deparaffinization through xylene, alcohol and distilled water, endogenous peroxidase was blocked by 3% H_2_O_2_ for 30 min. Sections were microwaved in 0.1 mM citrate buffer pH 6.0 for 20 min., and transferred to distilled water. After rinsing in phosphate‐buffered saline (PBS), sections were incubated with 1:10 normal donkey serum in PBS/4% BSA and then incubated overnight at 4°C with goat anti‐human MXRA5. Next, they were incubated with biotinylated rabbit anti‐goat antibody (1:300) in PBS–4% BSA for 30 min. at room temperature. After three rinses in PBS, they were incubated with streptavidin–peroxidase (Dako, Denmark) 1:500 for 30 min. Colour was developed with diaminobenzidine and then counterstained with haematoxylin, dehydrated and mounted with Canadian balsam (Polysciences). Specificity was checked by omission of primary antibodies and use of non‐immune sera.

Human kidney urine and tissue was obtained from the IIS‐Fundación Jiménez Díaz Biobank. Table S1 shows a clinical characterization of patients.

### Database search

The following databases were searched for MXAR5: Ensembl, ClinVar, GEO profiles (http://www.ncbi.nlm.nih.gov/geoprofiles/?term=MXRA5+kidney), Kidney & Urinary Pathway Knowledge Base (kupkb at http://www.kupkb.org/) and nephromine (http://www.nephromine.org/).

### Statistics

Statistical analysis was performed with SPSS 11.0 statistical software. Results are expressed as mean ± SEM for protein and mRNA expression experiments. Significance at the *P* < 0.05 level was assessed by Student's *t*‐test for two groups of data and anova for three or more groups.

## Results

### MXRA5 is up‐regulated in human kidney disease

**Figure 1:**
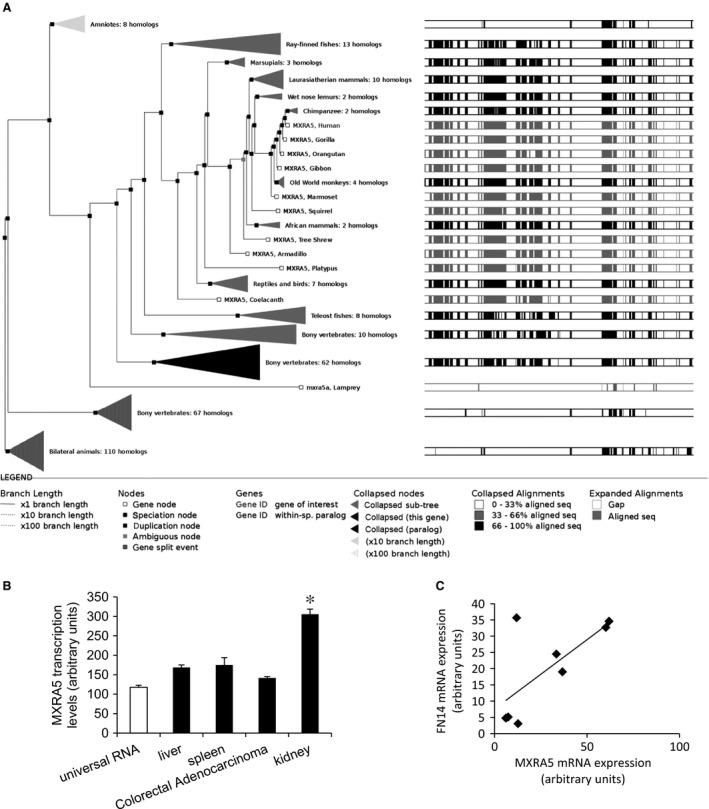
The MXRA5 gene. **(A)** Gene tree representing MXRA5 orthologues. The display shows the maximum likelihood phylogenetic tree representing the evolutionary history of gene. Note the existence of a human gene (Ensembl: accessed on January 25th). **(B)** MXRA5 gene expression in diverse human organs according to publicly accessible databases. Note high kidney MXRA5 mRNA levels as compared to other organs (*n* = 10, *P < 0.001 *versus* Universal RNA; source: GEO 17426686, 4689086). **(C)** Correlation between MXRA5 and Fn14 (TNFRSF12A) mRNA expression in human focal segmental glomerulosclerosis according to publicly accessible databases. Source: Nephromine 21.

MXRA5 is an evolutionary conserved gene. Although absent from mouse and rat, MXRA5 is expressed in a wide range of human organs and especially, in the normal human kidney (Fig. 1). The human kidney has relatively high levels of MXRA5 mRNA as compared to other organs 20 (Fig. 1B) (Table 1). In publicly available databases, MXRA5 was found to be up‐regulated in several forms of CKD, such as focal segmental glomerulosclerosis (FSGS), diabetic nephropathy, lupus nephritis, rapidly progressing glomerulonephritis and dysfunctional transplanted kidneys as well as in clear cell sarcoma, while there was no change in nephrosclerosis or renal cell carcinoma (Table 2 **)**. Furthermore, the kupkb database reflected the finding of MXRA5 in exosomes from normal urine in the Urinary Exosome Protein Database (http://dir.nhlbi.nih.gov/papers/lkem/exosome/). According to Nephromine, in FSGS, the nephropathy with highest MXAR5 expression, the best correlate of MXRA5 expression was the expression of the TWEAK receptor, Fn14 (correlation 0.713) (Fig. 1C) 21.

**Table 1:** MXRA5 expression in different human tissues

	**n‐fold**	***P*** **‐value**	**Reference**
Kidney *versus* liver	1.57	0.009	44
Kidney *versus* heart	1.34	0.033	44
Kidney *versus* liver	3.12	0.001	GEO: GDS1663/209596
Kidney *versus* spleen	2.06	0.001	GEO: GDS1663/209596

**Table 2:** MXRA5 mRNA expression in human kidney disease according to publicly available transcriptomics datasets

**Group**	**n‐fold**	***P*** **‐value**	**Reference**
Clear cell sarcoma of the kidney *versus* control	4.79	0.003	45
Focal segmental glomerulosclerosis *versus* normal kidney/minimal change disease	4.58	0.001	21
Diabetic nephropathy *versus* healthy living donor (glomeruli)	1.96	0.05	46
Lupus Nephritis *versus* healthy living donor (tubulointerstitium)	1.75	0.043	47
Diabetic nephropathy *versus* minimal change disease	1.53	0.029	48
Rapidly progressive glomerulonephritis *versus* healthy living donor/minimal change disease (glomeruli)	1.48	0.018	49
Diabetic nephropathy *versus* healthy living donor/minimal change disease	1.47	0.008	49
Renal dysfunction *versus* healthy living donor	1.24	0.003	50

No significant differences were found for nephrosclerosis: glomeruli *versus* healthy glomeruli from tumour‐free nephrectomy specimens 51 or for renal cell carcinoma *versus* adjacent non‐tumour tissue 52.

**Figure 2:**
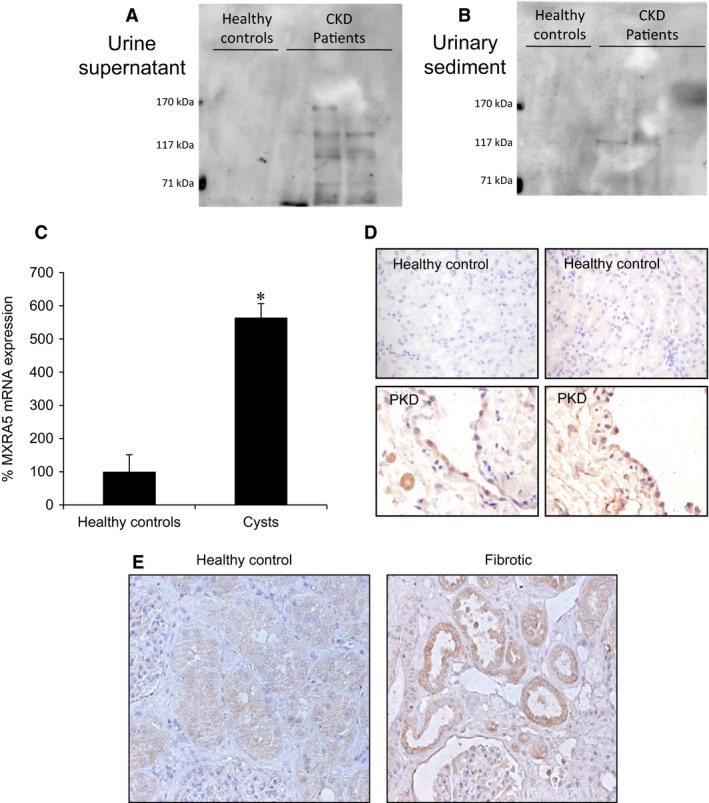
MXRA5 is present in CKD patients. (**A**) Urine supernatant. (**B**) Urine sediment. Western blot shows bands recognized by anti‐MXRA5 antibody in urine of CKD patients but not in healthy controls (*N* = 2 healthy controls and 3 CKD patients). (**C**) MXRA5 mRNA expression in ADPKD cysts or normal kidneys assessed by RT‐qPCR (*N* = 3 controls and 6 ADPKD patients, *P < 0.001 *versus* control). (**D**) MXRA5 immunohistochemistry in ADPKD cysts or normal kidneys (*N* = 6 controls and 6 ADPKD patients). Representative image. (**E**) MXRA5 immunohistochemistry in renal fibrosis and control kidney (*N* = 2 controls and 1 fibrotic, non‐ADPKD kidney).

Consistent with transcriptomics databases, proteins immune‐reactive for anti‐MXRA5 antibody were found both in the supernatant and sediment of centrifuged unconcentrated urine in CKD patients (samples 1‐3; Table S1), but not in healthy controls (samples 4 and 5; Table S1) (Fig. 2A and B). Furthermore, MXRA5 mRNA expression was increased in cysts from autosomal dominant polycystic kidney disease (ADPKD) patients (Fig. 2C) and expression was localized to cyst lining tubular cells by immunohistochemistry (Fig. 2D). Immunohistochemistry also localized MXRA5 staining to tubular cells in non‐ADPKD kidney fibrosis (Fig. 2E, corresponding to samples 16–18; Table S1).

### TGFβ1 and paricalcitol regulate MXRA5 expression in cultured human tubular cells

**Figure 3:**
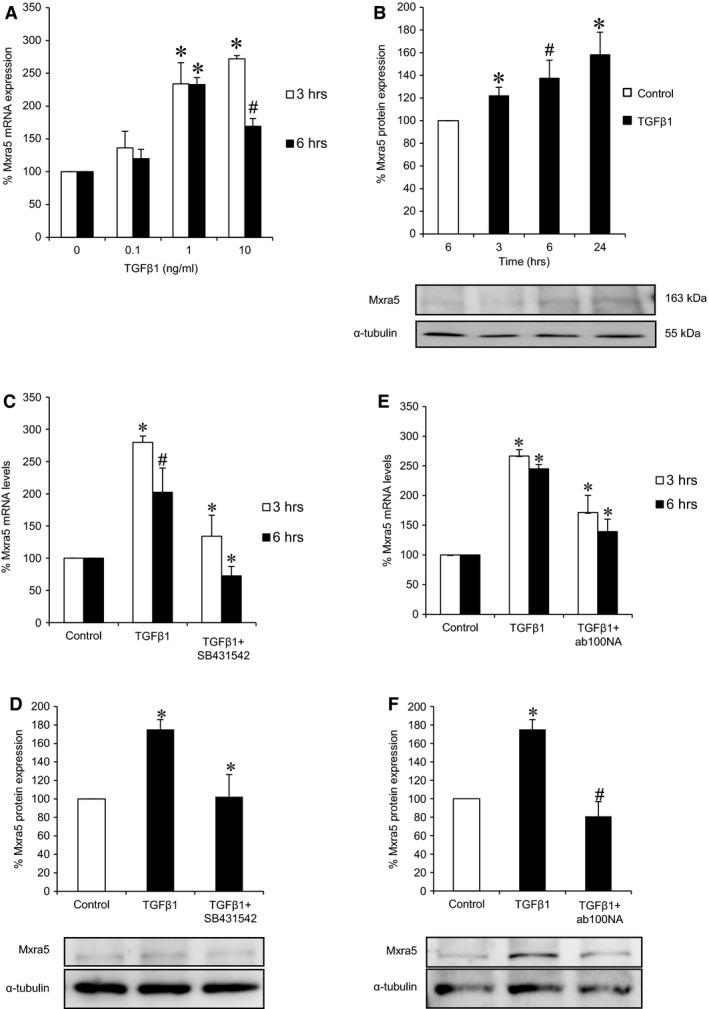
TGFβ1 increases MXRA5 in cultured proximal tubular cells. (**A**) Human proximal tubular cells were exposed to 0.1, 1 and 10 ng/ml TGFβ1 for 3 and 6 hr and MXRA5 mRNA expression was assessed by RT‐qPCR (*N* = 3, *P < 0.001 *versus* control, #*P* < 0.01 *versus* control). (**B**) Cells were exposed to 1 ng/ml TGFβ1 for 3, 6 and 24 hr and MXRA5 protein expression was assessed by Western blot. Tubulin was used as loading control (*N* = 3, *P < 0.025 *versus* control, #*P* < 0.05 *versus* control). (**C**) Cells were pre‐treated with 10^−5^ M TGFβ1 receptor 1 inhibitor SB431542 for 1 hr and then exposed to 1 ng/ml TGFβ1 for 3 and 6 hr, MXRA5 mRNA expression was assessed by RT‐qPCR (*N* = 3, *P < 0.001 *versus* control, #*P* < 0.006 *versus* control). (**D**) Cells were pre‐treated with 10^−5^ M SB431542 for 1 hr and then exposed to 1 ng/ml TGFβ1 for 6 hr, MXRA5 protein levels were assessed by Western blot (*N* = 3, *P < 0.005 *versus* control). (**E**) Cells were pre‐treated with 1 ng/ml neutralizing anti‐TGFβ1 antibody ab100NA for 1 hr and then exposed to 1 ng/ml TGFβ1 for 3 and 6 hr, MXRA5 mRNA expression was assessed by RT‐qPCR (*N* = 3, *P < 0.001 *versus* control). (**F**) Cells were pre‐treated with 1 ng/ml neutralizing anti‐TGFβ1 antibody ab100NA for 1 hr and then exposed to 1 ng/ml TGFβ1 for 6 hr, MXRA5 protein levels were assessed by Western blot (*N* = 3, *P < 0.005 *versus* control, #*P* < 0.018 *versus* control).

**Figure 4:**
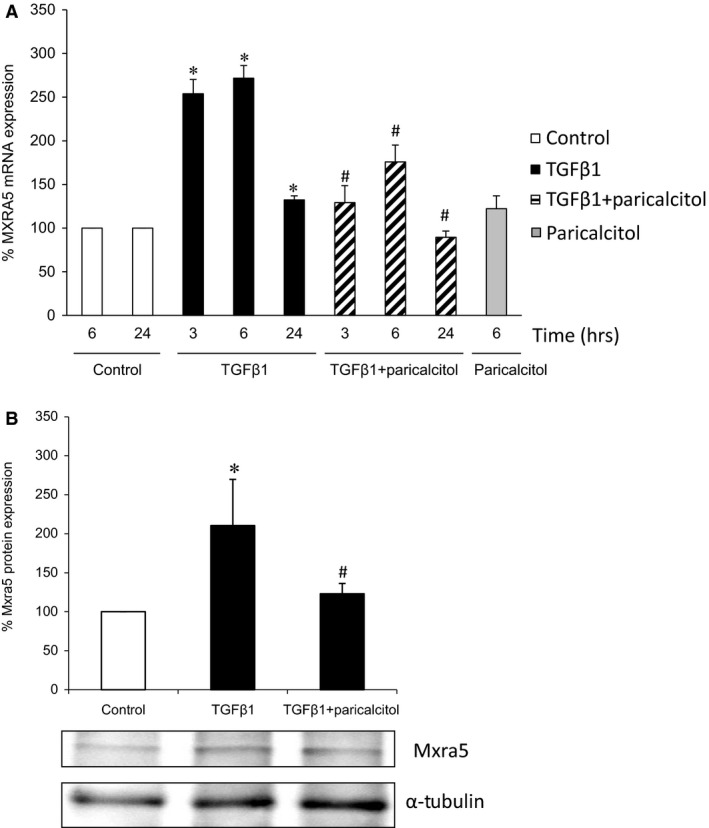
Paricalcitol prevents TGFβ1‐induced MXRA5 up‐regulation. Cells were pre‐treated with 1 μg/ml paricalcitol for 90 min. and then exposed to 1 ng/ml TGFβ1 for 6 hr. (**A**) MXRA5 mRNA expression was assessed by RT‐qPCR (*N* = 3, *P < 0.001 *versus* control, #*P* < 0.018 *versus* control). (**B**) MXRA5 protein expression was assessed by Western blot (*N* = 3, *P < 0.02 *versus* control, #*P* < 0.05 *versus* control).

After observing an up‐regulation of MXAR5 expression in human nephropathies characterized by kidney inflammation and fibrosis, we explored the potential regulation of MXRA5 expression by a representative fibrogenic cytokine TGFβ1 and a representative pro‐inflammatory cytokine, TWEAK. Therapies targeting TWEAK and TGFβ1 are under clinical development. Despite the good correlation between MXRA5 and Fn14 expression in FSGS, TWEAK did not modulate MXRA5 mRNA levels in tubular cells (Fig. S1). MXRA5 being an ECM protein, we next tested its regulation by TGFβ1. TGFβ1 dose‐dependently up‐regulated MXRA5 mRNA expression (Fig. 3A). The concentration of 1 ng/ml TGFβ1 was chosen for further studies. Stimulation of tubular cells with 1 ng/ml TGF‐β1 up‐regulated MXRA5 protein levels in whole cells in a time‐dependent manner (Fig. 3B) TGFβ1‐induced MXRA5 up‐regulation was prevented by the TGFβ1 receptor 1 (ALK5) inhibitor SB431542 (Fig. 3C and D) and by the neutralizing anti‐TGFβ1 antibody ab100NA (Fig. 3E and F). Vitamin D has been shown to regulate kidney fibrosis. The vitamin D receptor activator paricalcitol also inhibited the increase in MXRA5 mRNA (Fig. 4A) and protein expression (Fig. 4B) induced by TGFβ1.

### MXRA5 has anti‐inflammatory and anti‐fibrotic properties in cultured tubular cells

**Figure 5:**
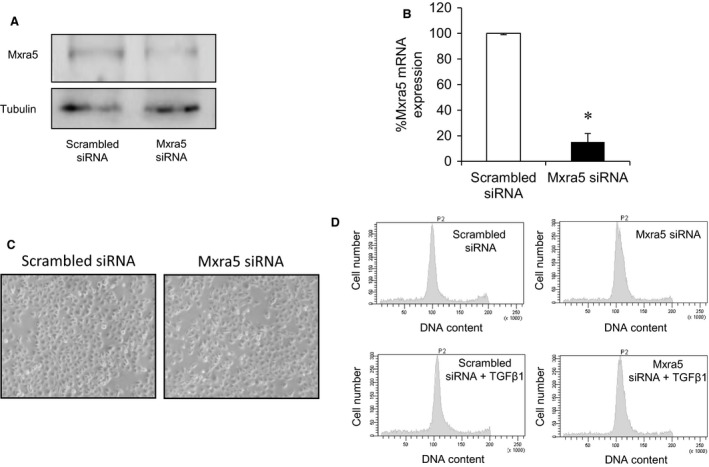
MXRA5 targeting has no effect on cell viability or proliferation. MXRA5 was successfully knocked down by means of a specific siRNA. (**A**) MXRA5 protein expression was assessed by Western blot (**B**) and RT‐qPCR (*P < 0.001 *versus* control). (**C**) No morphological changes were observed in MXRA5 knocked down cells. Contrast phase microscopy. (**D**) No changes in cell death or proliferation were observed in MXRA5‐silenced cells. DNA was stained with propidium iodide in permeabilized cells and DNA content quantified by flow cytometry.

MXRA5 was knocked down by means of specific MXRA5 siRNA (Fig. 5A and B). Down‐regulation of MXRA5 did not alter the morphological appearance of cells for up to 72 hr (Fig. 5C), and changes in cell cycle or cell death were not observed upon MXRA5 down‐regulation (Fig. 5D).

**Figure 6:**
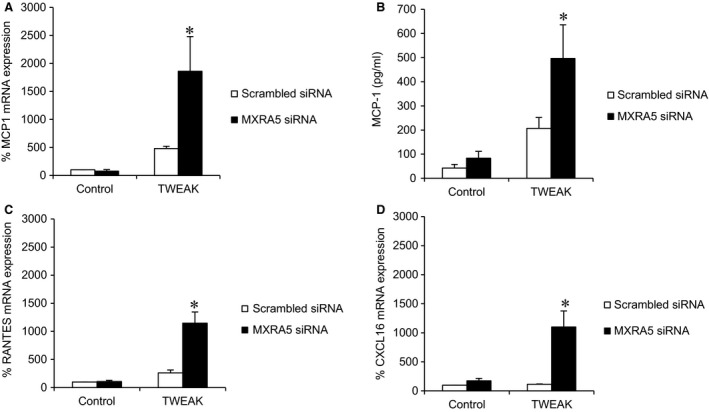
Endogenous constitutive MXRA5 has an anti‐inflammatory role in cultured proximal tubular cells. MXRA5 was knocked down and then cells were treated with 100 ng/ml TWEAK for 3 hr. TWEAK did not modify MXRA5 expression (supplemental figure). (**A**) MCP1 mRNA expression assessed by RT‐qPCR (*N* = 3, *P < 0.002 *versus* control). (**B**) MCP1 secretion assessed by ELISA in cell supernatants (*N* = 3, *P < 0.025). (**C**) Rantes mRNA expression assessed by RT‐qPCR (*N* = 3, *P < 0.001 *versus* control). (**D**) Cxcl16 mRNA expression assessed by RT‐qPCR (*N* = 3, *P < 0.001 *versus* control).

**Figure 7:**
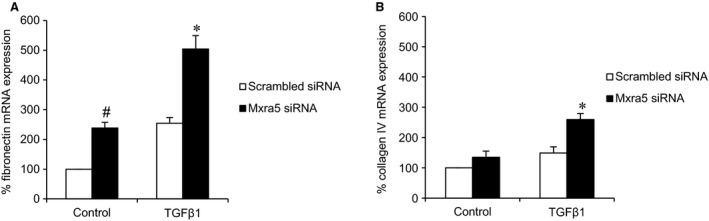
MXRA5 has an anti‐fibrotic role in cultured proximal tubular cells. MXRA5 was knocked down and then cells were treated with 1 ng/ml TGFβ1 for 6 hr. (**A**) Fibronectin mRNA expression assessed by RT‐qPCR (*N* = 3, *P < 0.001 *versus* control, #*P* < 0.005 *versus* control). (**B**) Type IV collagen mRNA expression assessed by RT‐qPCR (*N* = 3, *P < 0.002 *versus* control).

As previously described, TWEAK elicited pro‐inflammatory responses in tubular cells [12, 22]. Down‐regulation of MXRA5 resulted in increased expression of chemokine mRNA in response to TWEAK stimulation (Fig. 6), as well as in increased MCP‐1 protein levels in cell supernatants (Fig. 6B), suggesting that endogenous constitutive MXRA5 may play an anti‐inflammatory role. TGFβ1 is a key fibrogenic cytokine in tubular cells and kidney injury 23. MXRA5 down‐regulation resulted in increased expression of genes encoding the ECM proteins fibronectin and type IV collagen in response to TGFβ1 stimulation (Fig. 7), suggesting that TGFβ1‐induced MXRA5 expression contributes to limit the fibrogenic response of tubular cells. These results suggest that constitutive or inducible MXRA5 has anti‐inflammatory and anti‐fibrotic properties.

### MXRA5 in kidney cancer

**Figure 8:**
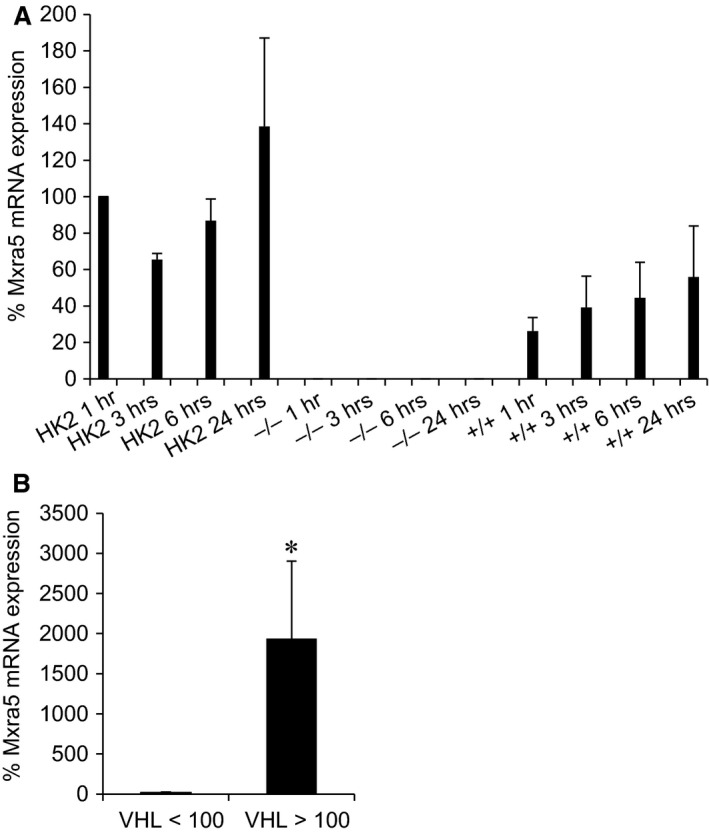
MXRA5 in cancer. (**A**) Human proximal tubular cells and VHL ^−/−^ and VHL ^+/+^ clear cell carcinoma cells were serum‐depleted and collected at 1, 3, 6 and 24 hr. MXRA5 mRNA expression was assessed by RT‐qPCR (*N* = 3). (**B**) MXRA5 and VHL mRNA expression assessed in biopsies from renal carcinoma tissue (*N* = 10) and healthy kidney tissue (*n* = 9) from the same kidney (*P < 0.05).

As MXRA5 is overexpressed in ovarian, lung and colorectal cancer 24, 25, 26, we studied MXRA5 expression in VHL‐defective ccRCC cell line (VHL^−/−^). MXRA5 mRNA expression was assessed in untreated HK2 cells and VHL^−/−^ cells, as well as VHL‐expressing ccRCC cells (VHL^+/+^) (Fig. 8A). No MXRA5 mRNA expression was observed in VHL^−/−^ cells, suggesting a relationship between MXRA5 and tumourigenesis. We then studied MXRA5 expression in renal biopsies from renal carcinoma patients. We observed a direct relationship between VHL and MXRA5 transcriptional expression (Fig. 8B). Thus, those patients with low VHL expression showed low MXRA5 expression, as observed for cultured ccRCC.

## Discussion

The main finding of our study is that MXRA5 is an anti‐inflammatory and anti‐fibrotic molecule up‐regulated by the fibrogenic cytokine TGFβ1 in human proximal tubular cells. This knowledge may be used to develop novel therapies for CKD.

CKD is characterized by inflammation and accumulation of extracellular matrix and TWEAK and TGF‐β1 were chosen as representative pro‐inflammatory and fibrogenic cytokines respectively. Tubular cells are key cells in tubulointerstitial inflammation. In response to an adverse microenvironment, tubular cells are activated to express chemotactic, pro‐inflammatory and pro‐fibrotic molecules, including cytokines such as TNF, Fas ligand, TRAIL and TWEAK and chemokines such as MCP‐1, RANTES and CXCl16 that decisively contribute to recruit inflammatory cells and to promote kidney injury [12, 22, 27, 28]. In this regard, tubulointerstitial damage correlates better with outcome of renal function than glomerular injury scores, even for primary glomerulonephritis 29. Indeed, tubulointerstitial fibrosis is considered an important predictor of renal survival 30 and TGFβ1 is a key pro‐fibrotic cytokine that contributes to renal fibrosis 31, 32, 33, while TWEAK is a key pro‐inflammatory cytokine which promotes glomerular and tubulointerstitial inflammation and fibrosis 12. Up‐regulation of TGF‐β1 is a universal finding in CKD, both in animal models and in humans. Inhibition of TGFβ1 by multiple strategies suppresses renal fibrotic lesions and prevents progressive loss of kidney function 34. Interestingly, TGFβ1 also has anti‐inflammatory actions. Indeed, TGF‐β1 KO mice display a systemic inflammatory disease, causing, among others, kidney inflammation 35. We have now observed that TGFβ1, but not the inflammatory cytokine TWEAK, up‐regulated MXRA5 expression in tubular cells through the activation of the TGFR1 receptor. This suggests that MXRA5 might mediate some TGFβ1 actions. In this regard siRNA targeting of MXRA5 led to a striking increase in the pro‐inflammatory response to TWEAK. Thus, MXRA5, as TGFβ1, behaves as an anti‐inflammatory molecule in tubular cells. By contrast, MXRA5 targeting magnified TGFβ1‐induced expression of ECM genes, suggesting that MXRA5 may also limit some TGFβ1‐elicited responses, such as fibrosis. Overall, the dual anti‐inflammatory and anti‐fibrotic activity of MXRA5 suggests that this is a molecule of potential therapeutic interest and that MXRA5 up‐regulation in CKD may be an adaptive response that breaks inflammation and fibrosis. In this regard, characterization of the MXRA5 receptor and generation of agonistic peptides may provide a new avenue for research in kidney disease. However, the fact that mice and rats do not express MXRA5 will complicate the development of preclinical models to address its *in vivo* function in kidney disease. The observation that the VDR activator paricalcitol prevents MXRA5 up‐regulation in response to TGFβ1 is especially interesting. Thus, paricalcitol has anti‐inflammatory and anti‐fibrotic properties that may compensate for the loss of the anti‐inflammatory and anti‐fibrotic action of MXRA5 [36, 37]. Thus, paricalcitol directly decreased the expression of cytokines and extracellular matrix and moderated Wnt/β‐catenin signalling and NF‐κB and Snail activation in cultured renal cells 36, 37, 38, 39, 40. While nephroprotective actions of VDR activation have been observed in numerous rat and mice models, human confirmation is lacking 41. Among the potential explanations for this discrepancy, we should now add the lack of MXRA5 in rats and mice used as models of kidney disease, while this potentially nephroprotective molecule is present in humans and down‐regulated by VDR activators. Thus, we speculate that the coexistence of nephroprotective and non‐nephroprotective actions of paricalcitol may be one of the contributing factors to the clinical failure of paricalcitol in kidney disease.

Urine contained anti‐MXRA5 immunoreactive peptides that may correspond to proteolytic fragments of MXRA5. Their presence in urine from CKD patients may point to a potential role as a biomarker. However, given the diverse sizes of the bands observed, this result should be considered preliminary. In this regard, increased tubular cell expression of MXRA5 was observed in human CKD tissue. The finding of MXRA5 in normal urine exosomes 42 may account for its presence in urine and might even contribute to its biological activity.

The present findings may also be relevant for cancer. MXRA5 was found overexpressed in ovarian cancer compared with normal ovaries and it was involved in tumour angiogenesis 24. It is also among the most frequently mutated genes in non‐small cell lung carcinomas 25. MXRA5 was proposed as a novel biomarker in colorectal cancer 26, as it was overexpressed in colorectal cancer tissue compared with their corresponding normal tissue [26, 43]. Our results further show that VHL may be a driver of MXRA5 expression in cancer cells.

In conclusion, MXRA5 is a molecule involved in the regulation of important biological processes, such as inflammation and fibrosis, during kidney disease. Additional studies will be required to determine the clinical relevance of MXRA5 as a biomarker in renal disease as well as to better characterize the molecular determinants of MXRA5 action, including the search for receptors.

## Supporting information


**Figure S1** TWEAK does not modulate MXRA5 expression.

**Table S1** Clinical characteristics of patients
