# Supplementary figures and images for: MXRA5 is a TGF‐β1‐regulated human protein with anti‐inflammatory and anti‐fibrotic properties
Source: J Cell Mol Med. 2016 Sep 6;21(1):154–64. doi: 10.1111/jcmm.12953 (PMC5192817; doi:10.1111/jcmm.12953)

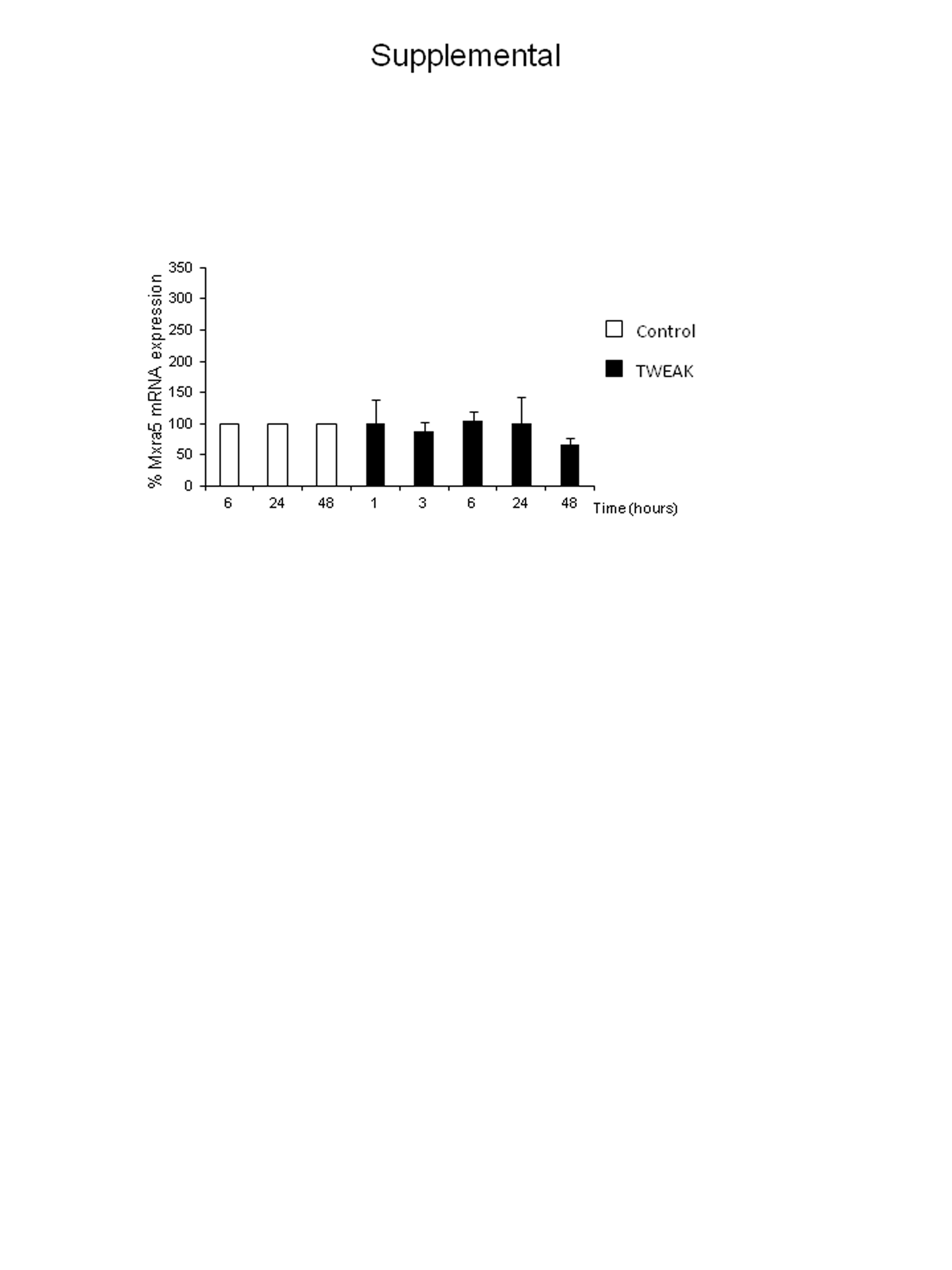

Supplement: Supplementary file 1 — Figure S1 TWEAK does not modulate MXRA5 expression. [file JCMM-21-154-s001.tif]
