# Supplementary material for: MXRA5 is a TGF‐β1‐regulated human protein with anti‐inflammatory and anti‐fibrotic properties
Source: J Cell Mol Med. 2016 Sep 6;21(1):154–64. doi: 10.1111/jcmm.12953 (PMC5192817; doi:10.1111/jcmm.12953)
Supplement: Supplementary file 2 — Table S1 Clinical characteristics of patients [file JCMM-21-154-s002.docx]

**Supplementary table 1. Clinical characteristics of patients.**

| Patient | Sex | Age (years) | eGFR (ml/min/1.73 m^2^) | Sample |
| --- | --- | --- | --- | --- |
| 1 | Female | 78 | 76 | Urine |
| 2 | Male | 34 | 54 | Urine |
| 3 | Male | 62 | 123 | Urine |
| 4 | Male | 25 | 64 | Urine |
| 5 | Female | 27 | 160 | Urine |
| 6 | Male | 77 | 91 | Kidney tissue |
| 7 | Male | 63 | 100 | Kidney tissue |
| 8 | Female | 67 | 66 | Kidney tissue |
| 9 | Female | 59 | 100 | Kidney tissue |
| 10 | Male | 53 | 11 | Kidney tissue |
| 11 | Female | 79 | 92 | Kidney tissue |
| 12 | Female | 40 | 80 | Kidney tissue |
| 13 | Male | 83 | 79 | Kidney tissue |
| 14 | Female | 45 | 54 | Kidney tissue |
| 15 | Male | 58 | 9 | Kidney tissue |
| 16 | Male | 74 | 41 | Kidney tissue |
| 17 | Male | 77 | 91 | Kidney tissue |
| 18 | Male | 77 | 28 | Kidney tissue |
| 19 | Male | 47 | 58 | Kidney tissue |
| 20 | Female | 58 | 101 | Kidney tissue |
| 21 | Male | 63 | 90 | Kidney tissue |
| 22 | Female | 52 | 104 | Kidney tissue |
| 23 | Female | 59 | 94 | Kidney tissue |
| 24 | Female | 67 | 58 | Kidney tissue |
| 25 | Female | 64 | 91 | Kidney tissue |
| 26 | Male | 56 | 83 | Kidney tissue |
| 27 | Female | 73 | 90 | Kidney tissue |
| 28 | Female | 52 | 104 | Kidney tissue |
